# The effects of aquarium culture on coral oocyte ultrastructure

**DOI:** 10.1038/s41598-018-33341-x

**Published:** 2018-10-11

**Authors:** Chiahsin Lin, Jian-Ming Zhuo, Gabriella Chong, Li-Hsueh Wang, Pei-Jie Meng, Sujune Tsai

**Affiliations:** 1 National museum of Marine Biology & Aquarium, 2 Houwan Road, Checheng, Pingtung, 944 Taiwan; 2 Institute of Marine Biology, National Dong Hwa University, 2 Houwan Road, Checheng, Pingtung, 944 Taiwan; 3 Department of Biotechnology, Mingdao University, 369 Wen-Hua Road, Peetow, ChangHua, 52345 Taiwan; 4 Department of Post Modern Agriculture, Mingdao University, 369 Wen-Hua Road, Peetow, Chang Hua, 52345 Taiwan

**Keywords:** Conservation biology, Animal physiology

## Abstract

As the world’s oceans are currently threatened by anthropogenic pollution and climate change, coral breeding has become an important conservation method, since it can limit marine organisms’ exposure to sub-optimal environment conditions. However, the aquarium environment is inherently different from the ocean, and this could manifest in physiological changes in the reared organisms, particularly with respect to their reproduction. Therefore, the aim of this study was to observe and compare the ultrastructure of the oocytes from wild *Oxypora lacera* and *Echinopora gemmacea* with the oocytes from cultured corals using transmission electron microscope. The oocytes from Wild *O. lacera* and *E. gemmacea* were larger than cultured ones, though their microvillus layers were significantly thiner. Internally, lipid granule areas and yolk material density in the oocytes of wild *O. lacera* and *E. gemmacea* were ~25% lower than in their cultured counterparts. Food availability and the presence and availability of symbiotic dinoflagellates (genus *Symbiodinium*) may have played a role in driving these lipid-based differences, in particular, as cultured corals had limited potential for heterotrophic feeding. These data will aid in future coral husbandry efforts.

## Introduction

Coral reefs are common structures across Earth’s tropical seas; apart from their critical role in harbouring an immense degree of marine biodiversity^1,2^, they also serve as a natural barrier against storms and strong wave action^3^. Furthermore, they possess tremendous economic value given their importance in the tourism and fisheries industries^4^. Unfortunately, coral reefs areas are threatened by climate change, anthropogenic pollution, and destructive fishing practices^5–7^. As a result, reefs are deteriorating at an alarming rate, and coral bleaching, whereby the coral-dinoflagellate (genus *Symbiodinium*) endosymbiosis disintegrates, is becoming more commonplace on a global scale^8^. Ultimately, increased frequency of coral bleaching combined with the low fecundity associated with such sub-optimal environmental conditions^9–11^ may lead to species extinction. It is therefore imminent that the issue of reef degradation be addressed with conservation strategies, such as coral husbandry and cryopreservation of important coral genetic material, such as sperm and oocytes.

Although *in situ and ex situ* conservation strategies are generally regarded to be effective in marine conservation, they serve different purposes. The former include marine parks and aim to preserve marine organisms by protecting them in their natural habitats by controlling and/or limiting human activities^12,13^. While properly managed marine parks have successfully allowed for species to rebound, their lack of clear boundaries and typically large areas make them difficult to manage effectively^14^. Moreover, marine parks cannot save coral reefs from the effects of climate change^15^, dispersive pollutants^16,17^, or marine pathogens^18^. These issues do no impede *ex situ* conservation strategies, which were originally developed for the purposes of gene banking, education, tourism, and research but could be applied as a tool for conservation when individual species’ numbers become so low that intensive conservation efforts are required^19^. Corals have been successfully transplanted to artificial settings such as husbandries and nurseries for the purpose of captive breeding and mariculture for reef restoration^20^, and such coral nurseries can further provide a safe refuge for coral larvae when they are at their most vulnerable stage; transplantation back to reefs can then be carried out when corals are large enough to have a higher chance of survival, thus preventing species extinction^21^.

Although *ex situ* conservation is likely to become increasingly vital for the survival or certain species, *ex situ* conservation has its own shortcomings, such as the maintenance of the culture facilities, and the inherent, gradual loss of genetic diversity when organisms are reared in captivity over long-term timescales^22^. Sub-optimal husbandry conditions may inflict changes on the cultured organisms’ growth, reproductive capacity, and behaviour. Effectiveness and success of coral propagation, in particular, depends on light^23–25^, temperature^26^, nutrient and food availability^27^, water flow^28^, and water quality^29^. Also, in the initial culture period, corals may experience slower growth *ex situ* compared to their natural reef habitat^30^ possibly due to the energy expenditure required to acclimatize to the aquarium environment^31^.

For coral propagation, fragmentation has been a widespread practice, as it allows for rapid proliferation at a high survival rate^32^. However, to maintain genetic diversity, corals may also undergo sexual reproduction. Thus, it is equally important that corals maintain their fecundity in the aquarium setting^33^. The morphology and ultrastructure of oocytes have a critical role in fertilization success, as abnormalities could result in failed fertilization or retard coral larvae development. Previous studies by Tsai *et al*.^34,35^ and Padilla-Gamino *et al*.^36^ on coral oocyte ultrastructure have found that the majority of the volume of the oocytes consists of yolk materials such as yolk bodies, lipid granules, and corticol alveoli, which serve as energy reserves for the oocyte and aiding in maintaining buoyancy^37^. In addition to these yolk materials, high numbers of vesicles can also be found within the cytoplasm. These vesicles may serve as carriers for nutrient transfer or provide additional support for the developing blastocyst^34^. Mitochondria can also be found in the oocyte cytoplasm due to their main role in the supply of energy needed for oocyte function and development^38^. At the surface, the vitelline membrane envelopes the whole oocyte, including the microvilli and oocyte membrane. The vitelline membrane is equivalent to the zona pellucida of mammals, serves as a protective layer, and is responsible for the cortical reaction during fertilization^39^. The microvilli and oocyte membrane found beneath the vitelline layer also function as a protective barrier to the external environment.

All reef-building scleractinian corals maintain an endosymbiotic relationship with dinoflagellates of the genus *Symbiodinium*, and corals depend on organic compounds synthesized by these photosynthetically active *Symbiodinium* to fuel their growth, calcification^40^, and gamete production^41^. High *Symbiodinium* densities are therefore required to corals to maintain a high level of health^42^. Nonetheless, *Symbiodinium* density can change in response to environmental changes, and fluctuations in light, water temperature, and nutrient availability may affect their activity, rates of cell division, and density^43^. At high temperatures and light levels, *Symbiodinium* chloroplast may bleach, or, alternatively, the entire cell may be ejected from the coral host, both of which result in paling of the host tissues^44^. Acquisition of *Symbiodinium* into the coral gastrodermal cells is generally via either vertical or horizontal transmission. In the former process, *Symbiodinium* are transferred from parent to oocyte^45^ or larvae. These oocytes and larvae have the advantage of an additional food source upon which to rely during their development^46,47^ in contrast, horizontal transmission, whereby *Symbiodinium* are acquired directly from the water column gives the oocytes or larvae a change to uptake strains of *Symbiodinium* that may be better suited to their new environment^48,49^.

Both *O. lacera* and *E. gemmacea* are widely distributed across the reefs of Indo-West Pacific and could be found colonizing almost similar depth range. Both species were heavily influenced by the global warming and anthropogenic activities that affect their symbiotic relationship. Fertilization occurs within few hours after dispersion when eggs and sperms mix in water column. Unfertilized gametes gradually deteriorate over the next few hours after release from the parents. Nonetheless, there has been no comprehensive investigation of the gametogenic cycle specifically of *O. lacera* and *E. gemmacea* in the reef of Kenting, Taiwan. However according to our observation *O. lacera* spawned six to seven days after full moon. *E. gemmacea* spawned almost simultaneously to *O. lacera*. Spawning may last for two days as observed in the same study. In our experience, spawning had mostly occurred approximately two hours after sunset (1830 hours). The ensuing dataset could not only be of biological interest, but could also aid in the development of coral husbandry facilities and help to optimize cryopreservation protocols. Understanding the morphological changes in coral oocytes reared in captivity vs. conspecifics sampled *in situ* is critical since oocytes are responsible for the future growth and proliferation of the captive organisms. However, coral oocytes are sensitive to changes in seawater quality^50–52^. Therefore, the aim of the present study was to observe and compare the ultrastructure and morphology of both wild and reared oocytes of two hard coral species, *Oxypora lacera* and *Echinopora gemmacea*.

## Results

### Biological and morphological characteristics of coral oocytes

According to the light microscopic analysis, *O. lacera* and *E. gemmacea* oocytes were generally spherical in shape. *O. lacera* oocytes were orange in color, whereas *E. gemmacea* oocytes were deeppink. Oocytes of both species measuring 200–400 um in diameter were observed. The structure of both corals comprised a central core, gastrodermis and epidermis. Masses of white mesentery material were produced to separate oocytes from one another as well as to expel the oocytes during the final maturation stage.

### Morphological characteristic of *O. lacera* oocytes

**Figure 1:**
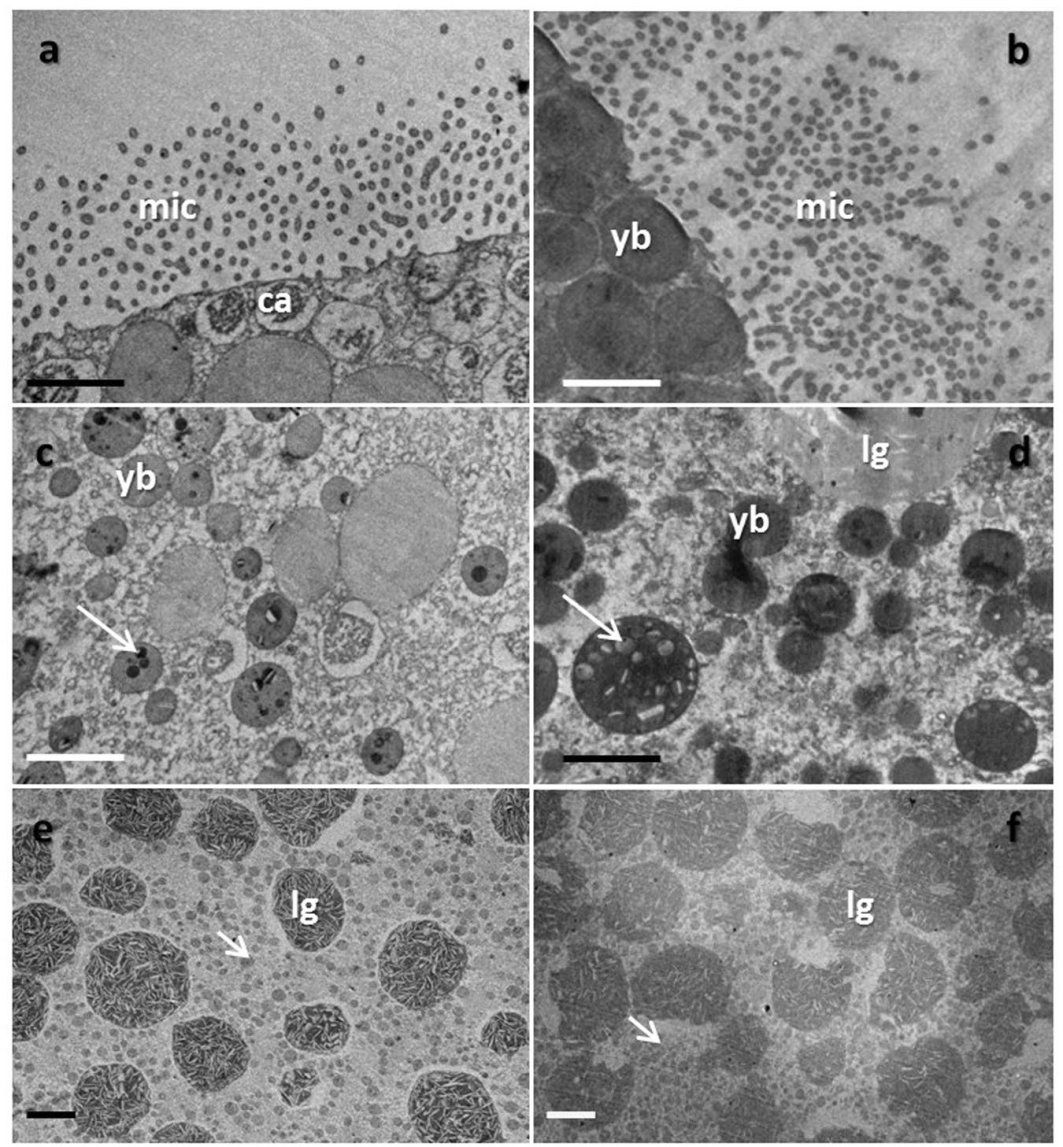
TEM images of wild (**a**,**c** and **e**) and cultured (**b**,**d** and **f**) oocytes of *Oxypora lacera*. Images were taken of microvilli (“mic;” **a**,**b**), yolk bodies (“yb;” **c**,**d**), and lipid granules (“lg;” **e**,**f**). The scale bars represent 1 and 5 um for panels a–d and e, f, respectively.

Microvilli were observed to surround oocytes from both the wild and cultured *O. lacera* (Fig. 1). Due to the spherical shape of the oocytes and the sectioning angle, the microvilli, which normally appear as finger-like projections, instead appeared as a series of dots surrounding the oocytes’ periphery (Fig. 1a,b). An observational comparison between the oocytes from wild and cultured corals (Table 1) revealed that microvilli of oocytes from wild corals were less dense and shorter that those oocytes from cultured corals (Fig. 1a). In the oocytes from cultured corals, microvilli were more densely packed and significantly thicker (*p* < 0.05; Fig. 1b). Beneath the microvilli layer, the cortical alveoli and yolk bodies were found to be aligned closely to the oocyte membranes in the oocytes from wild corals while the oocytes from cultured corals, only yolk bodies were positioned closely to the oocyte membranes (Fig. 1a,b).

**Table 1:** Morphological and ultrastructural data. Error terms represent standard deviation, and lowercase letters denote Tukey’s post-hoc test differences between individual means (p < 0.05).

Species-collection site	Thickness of Microvillus layer	Yolk body	Lipid granule	Mitochondria
Diameter of organelles in the oocyte (μm)				
*O. lacera* (wild)	2.21 ± 0.21^a^	1.09 ± 0.18	8.24 ± 1.46	1.19 ± 0.12
*O. lacera* (cultured)	3.02 ± 0.68^b^	1.31 ± 0.25	8.46 ± 1.84	NONE
*E. gemmacea* (wild)	1.18 ± 0.22^a^	1.16 ± 0.31	7.21 ± 0.89^a^	1.07 ± 0.16*
*E. gemmacea*(cultured)	1.93 ± 0.14^b^	1.28 ± 0.61	8.88 ± 0.51^b^	1.04 ± 0.06
**Average number per unit area**				
	Yolk body density		Lipid granule density	
*O. lacera* (wild)	31.87 ± 6.92		16.64 ± 3.81	
*O. lacera* (cultured)	33.99 ± 3.10		23.05 ± 3.02	
*E. gemmacea* (wild)	25.81 ± 6.45		20.18 ± 2.42	
*E. gemmacea* (cultured)	25.13 ± 3.14		23.62 ± 3.31	
**Percent of oocyte volume occupied**				
	Yolk body	Lipid granule	Yolk body + lipid granule	
*O. lacera* (wild)	24.9%	30.5%	55.4%	
*O. lacera* (cultured)	38.1%	43.1%	81.2%	
*E. gemmacea* (wild)	22.7%	27.4%	50.1%	
*E. gemmacea* (cultured)	26.4%	48.1%	74.5%	
**Oocyte diameter (μm)**				
*O. lacera* (wild)	389.32 ± 21.32^a^			
*O. lacera*	302.43 ± 13.75^b^			
*E. gemmacea* (wild)	268.91 ± 10.02^a^			
*E. gemmacea*	207.25 ± 16.72^b^			

*Only two *E. gemmacea* samples were analyzed for mitochondrial size (*n* = 2).

**Figure 2:**
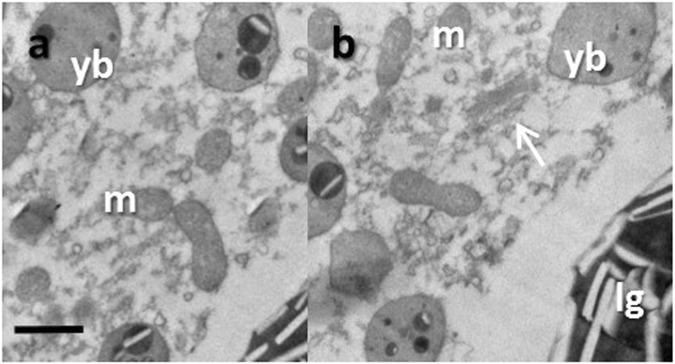
TEM images of *Oxypora lacera* mitochondria (m) and Golgi bodies (denoted by arrow) from wild (**a**) and cultured (**b**) oocytes. yb = yolk body. lg = lipid granule. The scale represents 1 um.

In the oocytes from both wild and cultured corals, the yolk bodies were spherically shaped, electron-dense, and granulated (indicated by arrows in Fig. 1c,d). Cultured oocyte yolk bodies were more electron-dense and larger (Table 1), while the yolk bodies of the wild oocyte were smaller and contained less granular material (Fig. 1c,d). Lipid granules were also found in abundance in the cytoplasm of oocytes from wild and cultured *O. lacera* (Fig. 1e,f). Lipid granules were differentiated from lipid bodies in that the former were larger, more spherical, and contained white strips of lipids. Again, the oocytes from cultured corals were observed to have higher density of lipid granules compared to the oocytes from wild corals (Fig. 1e,f and Table 1), though the difference was not statistically significant. Mitochondria were observed to be distributed near the yolk bodies and lipid granules in the oocytes from both wild and cultured corals. Mitochondrial shape was biconcave, discoid, and round due to the sectioning angle (Fig. 2a,b). Golgi bodies could readily be observed in close vicinity to the mitochondria, yolk bodies, and lipid granules in the oocytes from cultured corals (indicated by arrow in Fig. 2b), though they were less commonly seen in the oocytes from wild corals.

### Morphological characteristics of *E. gemmacea* oocytes

**Figure 3:**
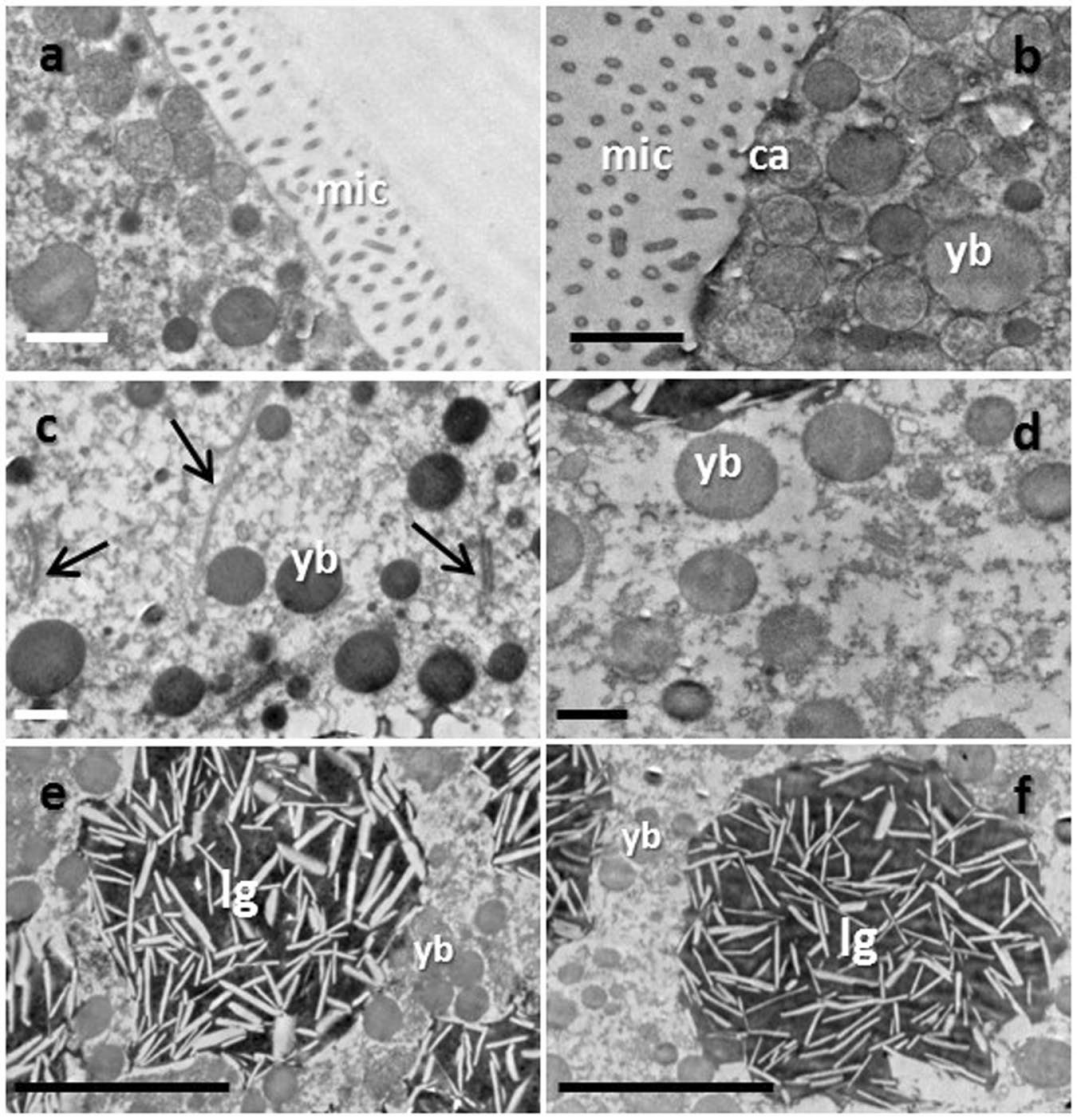
TEM images of wild (**a**,**c** and **e**) and cultured (**b**,**d** and **f**) *Echinopora gemmacea* oocytes. Images were taken of microvilli (“mic;” **a**,**b**), yolk bodies (“yb;” **c**,**d**), and lipid granules (“lg;” **e**,**f**). Golgi bodies are denoted by arrows in panel c. Ca = cortical alveoli. Scale bars represent 1 and 5 um for panels a–d and **e**,**f**, respectively.

**Figure 4:**
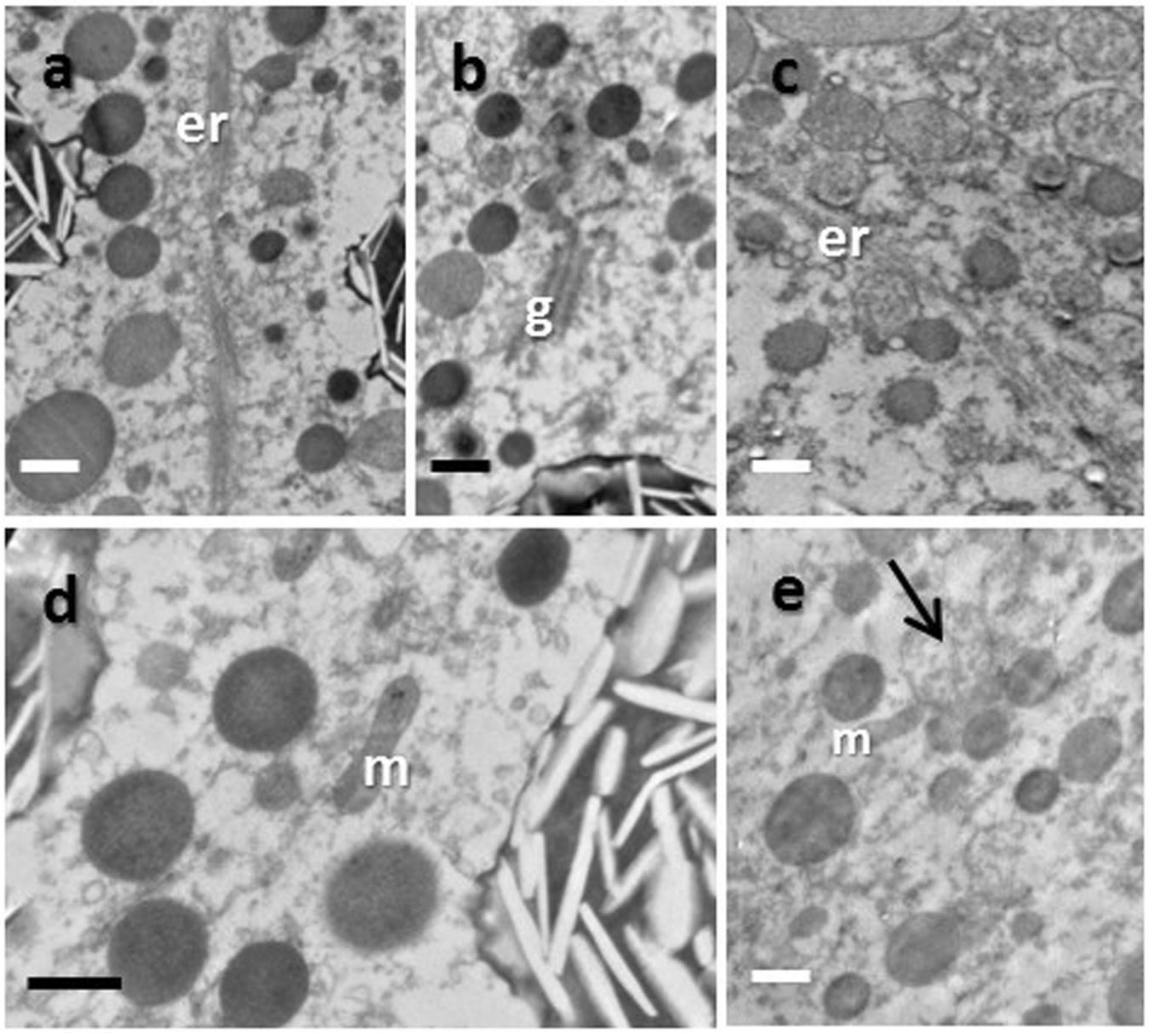
TEM images of wild (**a**,**b** and **d**) and cultured (**c** and **e**) *Echinopora gemmacea* oocytes. Mitochondria = m, Golgi bodies = g, endoplasmic reticulum = er. The black arrow in panel e marks a yolk inclusion. Scale bar = 1 um.

Oocytes of both wild and cultured *E. gemmacea* colonies were observed to be surrounded by microvilli (Fig. 3). Microvilli appeared as long, dotted strips in the TEM image due to the sectioning angle. A morphological comparison between the oocytes from cultured and wild corals revealed that the oocytes from wild corals have significantly thinner microvilli (Fig. 3a,b and Table 1). In the oocytes from both cultured and while corals, the microvilli were evenly spaced (Fig. 3a,b). Abundant cortical alveoli and yolk bodies were positioned near the oocyte membranes of both the wild and culture oocytes (Fig. 3c–f); yolk bodies were also found throughout the cytoplasm. In *E. gemmacea* oocytes, yolk bodies were agranular and homogeneous (Fig. 3c–f). The oocytes from wild corals were more electron-dense compared to cultured corals, but the yolk bodies of the oocytes from cultured corals were larger, and in higher abundance, compared to the oocytes from wild corals (Table 1). Lipid granules were found in abundance in oocytes of *E. gemmacea*, and their appearance was similar to those of *O. lacera*. Lipid granules of the oocytes from cultured corals were significantly larger than those of wild corals (Table 1). Other organelles, such as the endoplasmic reticula, Golgi bodies, and mitochondria were found near the yolk materials in the oocytes from both the wild and cultured corals (Fig. 4a–e).

### Comparison of the morphology of the oocytes from wild and cultured corals

**Figure 5:**
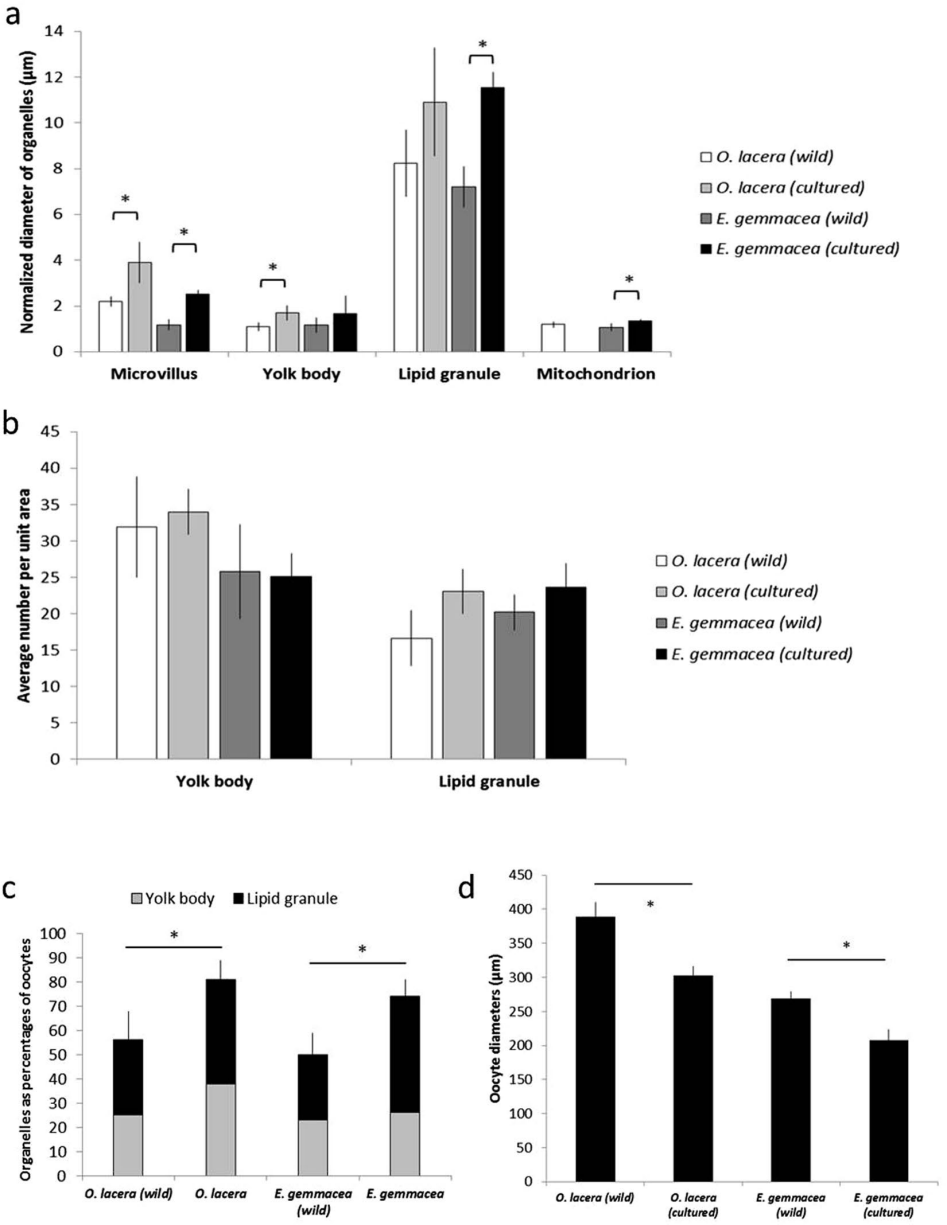
Oocyte morphology and ultrastructure for wild and cultured coral oocytes of two species: *Oxypora lacera* and *Echinopora gemmacea*. Normalized density of select organelles (**a**), organelle volume proportions (**b**), oocyte diameter (**c**) and organelle size (**d**) were measured in both wild and cultured oocytes of both species. Error bars represent standard deviation of the mean. When a significant difference was detected within species (wild vs. cultured; student’s *t*-test *p* < 0.05), the higher of the two values has been marked by an asterisk (*).

The size of organelles were measured according to the scale bar of each TEM image and by averaging the largest diameter in an image. The ultrastructure of both aquaria-reared and wild *O. lacera* and *E. gemmacea* oocytes were compared (Fig. 5). TEM revealed that microvillus layers of the oocytes from the wild were significantly thinnr for both species after normalizing with oocyte size (Fig. 5a). Internally, the oocytes were occupied by large numbers of organelles, such as Golgi bodies, mitochondria, yolk bodies, and lipid granules. Yolk bodies and lipid granules of both the wild *O. lacera* and *E. gemmacea* oocytes were smaller than those of the cultured corals (Fig. 5a); however, these differences only were statistically significant for *O. lacera*. In contrast, mitochondrial length was significantly different between the oocytes from cultured and wild *E. gemmacea* (Fig. 5a). In *O. lacera*, though, mitochondria were only observed in the wild oocytes.

In addition to these size differences, the average amount of yolk material per unit area differed between wild and cultured coral oocytes (Fig. 5b); specifically, the average numbers of yolk bodies and lipid granule in the wild oocytes of *O. lacera* and *E. gemmacea* were lower compared to the oocytes from cultured corals for both species; however, these differences were not statistically significant (Fig. 5b). Yolk material was the most abundant substance in both wild and cultured coral oocytes for both species, and wild coral oocytes had a significantly lower percentage of the oocyte volume occupied by yolk material (Fig. 5c); 55.4% (yolk body-24.9% + lipid granule-30.5%) in wild *O. lacera* oocytes vs. 81.2% (yolk body - 38.1%; lipid granule - 43.1%) in cultured ones. Wild and cultured yolk material percentage for *E. gemmacea* was 50.1 and 74.5%, respectively (Fig. 5c). The wild vs. cultured differences were statistically significant for both species. The diameters of wild oocytes of *O. lacera* (389.32 ± 21.32 µm) and *E. gemmacea* (268.91 ± 10.02 µm) were significantly larger than those of cultured *O. lacera* (302.43 ± 13.75 µm) and *E. gemmacea* (207.25 ± 16.72 µm), respectively (Fig. 5d). Finally, TEM analysis revealed that the oocytes from cultured and wild corals of both coral species did not inherit symbiotic dinoflagellates (genus *Symbiodinium*) from their parents.

### *Symbiodinium* density and protein concentration in corals

**Figure 6:**
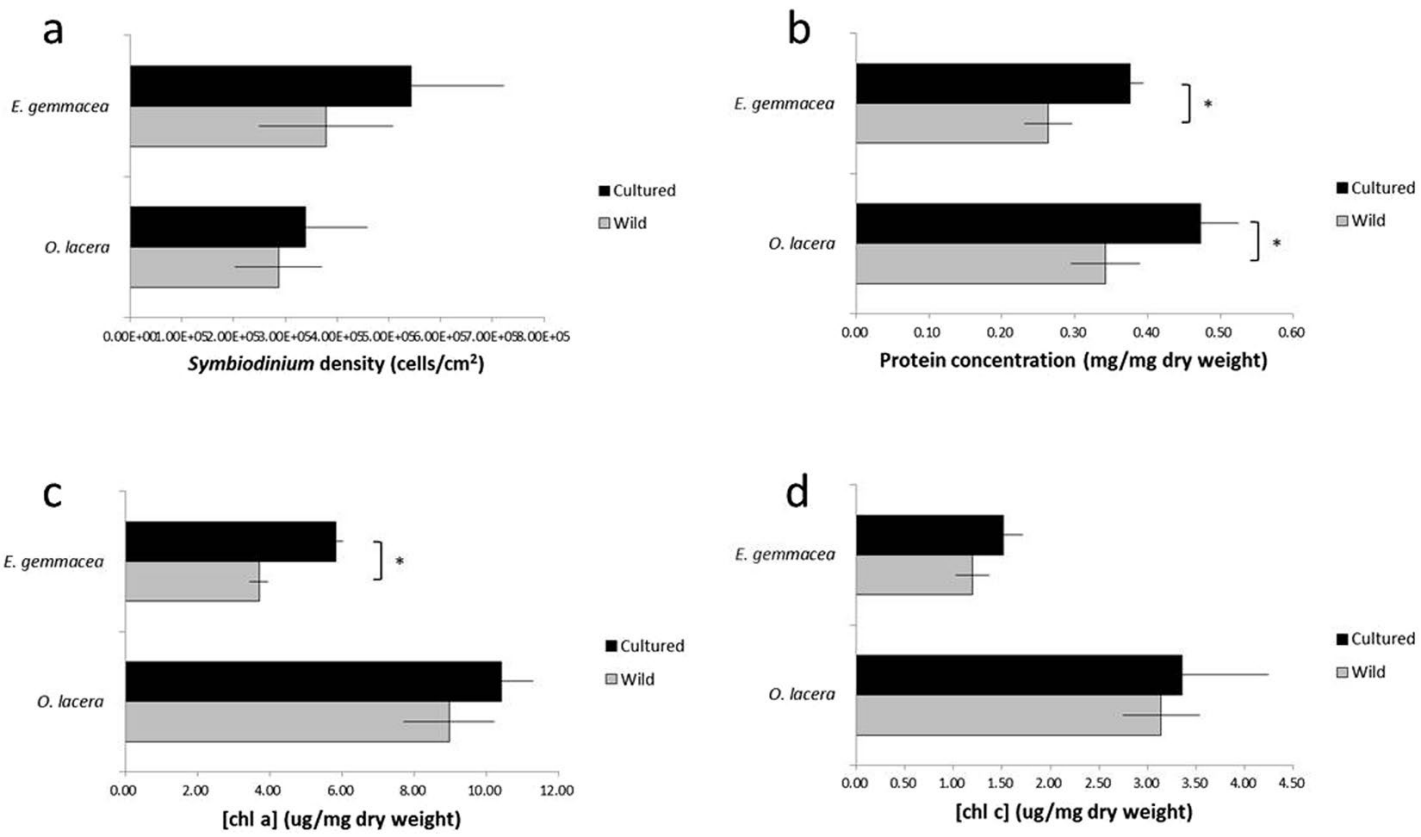
*Symbiodinium* analysis (**a**–**d**) in adult coral colonies: cell density (**a**), protein content (**b**), and chlorophyll a (Chl a) concentration (**c**,**d**). Error bars represent standard deviation (n = 3 replicate colonies for each treatment). When a significant difference (student’s *t*-test, *p* < 0.05) was detected between wild and cultured oocytes for a species, the higher of the two values has been marked with an asterisk (*).

Cultured coral colonies of *E. gemmacea* and *O. lacera* harboured more *Symbiodinium* (5.4 × 10^5^ and 3.4 × 10^5^ cells/cm^2^, respectively) compared to the two coral species in the wild (3.8 × 10^5^ and 2.9 × 10^5^ cells/cm^2^, respectively; Fig. 6a). Consequently, the higher *Symbiodinium* density within the cultured corals contributed to their higher chl *a* and *c* concentrations, as well as protein concentrations, than those in the wild coral colonies (Fig. 6b–d). Specifically, *Symbiodinium* density, chl *a* content, chl *c* content, and protein content were all higher in cultured coral for both target species.

### Water quality assessment

A comparison of water quality was made between the water from our sample site at Houbihu and the husbandry tanks at NMMBA (Table 2). From the results, no significant differences were detected in any physical (salinity, pH, and turbidity) or nutrient parameter (nitrite, nitrate, ammonia, phosphate, and silica).

**Table 2:** Water quality assessment in the husbandry facility and the site of oocyte collection. Error terms represent standard deviation of the mean. nd = not detected (<1 ug/L). Turb. = turbidity.

Station	Salinity	pH	[NO_3_]	[NO_2_]	[PO_4_]	[SiO_2_]	[NH_3_]	Turb.
	(*psu*)		(mg/L)	(mg/L)	(mg/L)	(mg/L)	(mg/L)	(*ntu*)
Oocyte collection Site	34.58 (0.03)	8.08 (0.01)	0.043 (0.012)	nd	nd	0.056 (0.006)	0.020 (0.002)	1.61 (0.54)
Husbandry facility	34.16 (0.21)	7.95 (0.06)	0.052 (0.006)	nd	nd	0.086 (0.002)	0.020 (0.004)	0.81 (0.13)

## Discussion

Oocytes that matured in experimentally reared organisms (i.e., *ex situ*) have been reported to be more competent that those collected *in situ*^53^, and coral spawning has previously been observed in other public aquaria; this suggests that sexual reproduction occurs in *ex situ*, albeit only in open, flow-through systems experiencing natural light levels^54^, Oocytes matured *ex situ* can still experience environmental variation with respect to light and nutrient availability, in particular. Both light and inorganic nutrients are essential to coral health, as *Symbiodinium* are photosynthetically active and contribute the majority of their fixed carbon to their coral hosts; the cnidarian hosts then use the energy derived for calcification^55^. Herein we did not observe variation in water quality between our husbandry tanks and the field, suggesting that the differences in oocyte quality cannot be directly attributed to water quality differences. A comparison between water quality data collected over eight years (2001–2008; [98]) with those of our husbandry tanks also revealed that seawater quality was similar with respect to the parameters measured. However, the lack of potential coral food sources in the aquarium’s seawater, which was not measured, may have affected the health of the corals. This idea is explored in greater detail elsewhere.

The present results indicate that the ultrastructure of both cultured *O. lacera* and *E. gemmacea* oocytes showed distinct differences in terms of morphology and size from the respective wild oocytes. Development of embryos is related to oocyte size^56^. In marine fish, large larvae derived from large oocytes can survive longer periods without feeding^57^, giving them a survival advantage. In the present study, wild oocytes were larger than cultured ones, suggesting that their overall quality and survivability are superior^56–58^.

Coral colony size may affect gamete production; smaller colonies are less fecund and also less likely to spawn due to the need for storing energy for other processes, such as growth^59^. Herein both *O. lacera* and *E. gemmacea* colonies in the NMMBA husbandry facility were found to spawn during each of the three survey years; however, they did not readily reproduce in the eight years prior to the start of this experiment. Although the actual reasons for this delay are unknown, we hypothesize several factors may be at play. First, NMMBA filters open ocean seawater through sand prior to its entry into the husbandry facility; this means that common coral food sources, such as phytoplankton, may not enter the husbandry tanks, forcing corals to rely almost solely on photosynthesis for energy. Furthermore, certain environmental factors that influence corals *in situ*, such as tidal levels^60^, do not vary *ex situ*^54^, and environmental variability has a strong impact on the physiology of Taiwanese corals^25^.

Finally, although the cultured corals were exposed to a natural light regime (albeit partially shaded by mesh tarps), light may still be obstructed by structures in the husbandry facility, such as the aforementioned the shading tarps. Since lunar irradiance dictates timing of coral spawning of the coral, obstructions such as, for instance, the walls of the tank, may cause differences in the light environmental that disrupt spawning behaviour. Furthermore, sub-optimal light intensity or quality in the husbandry setting have led to the smaller average size of the oocytes from cultured corals, since *Symbiodinium* photosynthetic efficiency can significantly influence coral fitness^24,61^ and reproduction^41,62^. Cultured corals were not fed, and NMMBA uses a sand filtration system to remove large particles, such as phytoplankton. Therefore, it is possible that the malnourishment of the parent colonies contributed to the small oocyte sizes in the cultured corals.

Finger-like projections known as microvilli are typically embedded in the vitelline layer of unfertilized coral oocytes and starfish embryos^34,63^. In the oocytes, microvilli may act as mechanical supports, and they facilitate nutrient uptake^64,65^. Microvilli are also involved in oocyte fertilization, and they hold blastomere cells together during the cleavage stage^63,66,67^. In the present study, microvillus layers were thicker in cultured coral oocytes of *O. lacera* and *E. gemmacea* compared to wild coral oocytes. Morphology and extension of the microvilli are controlled by the actin filaments located within them, and microvilli extend upon fertilization to hold the blastomere in place^63^. The thicker microvillus layers of the oocytes from cultured corals observed herein may be an adaptative feature; because microvilli also facilitate in nutrient uptake, we hypothesize that the thicker microvillus layers were to increase nutrient absorption in their hypothetically food-deprived environment (described above).

Similar to previous ultrastructure studies of marine invertebrate oocytes by Tsai *et al*.^35,65^ and Pipe^68^, the present study found that both type of *O. lacera* and *E. gemmacea* oocytes were occupied with large amount of yolk material, which encompasses yolk bodies and lipid granules. Yolk acts as an energy reservoir during oocyte maturation, fertilization, and embryo development^69^. Additionally, the yolk materials in coral oocytes facilitate buoyancy to increase their chances of fertilization success and dispersal^37,65^. The synthesis and metabolism of yolk material involves a range of organelles including Golgi bodies, mitochondria, and rough and smooth endoplasmic reticulum, hence their close proximity to each other in the ultrastructure images made herein and in Tsai *et al*.^70^.

Yolk materials were found at high densities/concentrations in the oocytes from *O. lacera* and *E. gemmacea* relative to wild oocytes^71^. Studies have showed that accumulation of yolk materials occurred due to *in vitro* incubation in sub-optimal culture media^72,73^. The results of these past studies collectively suggest that the high levels of lipids observed herein in the cultured coral oocytes may be a sign of cellular stress.

Simply having a large amount of yolk material is not necessarily indicative of oocyte stress. Pousis *et al*.^74^ reported that feeding contributed to the size differences in the yolk of wild and cultured Atlantic bluefin tuna *Thunnus thynnus* L. oocytes. The yolk of the captive-reared tuna oocytes was larger and denser than that of wild tuna oocytes, and the authors attributed the accumulation of yolk to the high-squid diet. As corals were not fed herein, we propose that the increase in lipid granules and yolk bodies in cultured coral oocytes can be attributed to *Symbiodinium* metabolism. *Symbiodinium* and fresh water algae *Scenedesmus* sp. accumulate lipids under nutrient-limited conditions^75–77^ Specifically, Muller-Parker *et al*.^78^ found that starvation of sea anemone hosts caused lipid accumulation in the *Symbiodinium in hospite*. Meanwhile, Jiang *et al*.^76^, Weng *et al*.^77^, and Li *et al*.^75^ all found that nitrogen limitation led to lipid accumulation. Jiang *et al*.^76^ suggested that the lipid accumulation by the dinoflagellates during periods of nitrogen deprivation is an adaptive response whereby the algae are actively increasing their energy stores for cellular processes necessitated to restore homeostasis in light of said starvation. Therefore, it is possible that the aforementioned lack of food in the cultured coral tanks led to the accumulation of lipids in the *Symbiodinium*, and these lipids were then translocated via the parent colony to the oocytes.

In regards to the functional differences between lipid granules and yolk body, there were no distinct functional differences as they act as energy storage and buoyance, to facilitate in oocyte fertilization and oocyte development. However, morphologically, lipid granules were larger in size compared to yolk bodies. Also, lipid granules were characterized by heterogeneous materials, white strips and spherical particles within the structure, while the yolk body appeared consisted of electron-dense homogenous material. This may be attributed to the difference in origin of these two organelles. Lipid granules may have been from synthesized from nutrients provided by *Symbiodinium* as suggested by Tsai *et al*.^35^ while formation of yolk bodies are associated to the rough endoplasmic reticulum and Golgi body^35,70^.

Cultured adult *O. lacera* and *E. gemmacea* colonies harbored higher densities of *Symbiodinium* than their wild-type counterparts did; accordingly, *Symbiodinium* metabolism possibly affected the oocyte quality and morphology. For instance, higher chlorophyll a and c concentrations might have increased the scope for photosynthesis and the subsequent translocation of fixed carbon into their coral hosts. Incubation at sub-saturating light levels can lead to increases in the chlorophyll concentration^79,80^, and in some cases, the *Symbiodinium* density as well^80,81^. This observation was in agreement with the present results because our corals were maintained in a semi-enclosed husbandry setting without artificial lighting and were subjected to obstruction by the building structure, thus resulting in decreased light irradiance. The phenomenon of an increased *Symbiodinium* number in coral colonies in deeper waters, which have lower light intensities and irradiance, has also been reported by Nir *et al*.^44^ and Battey and Porter^82^. Titlyanov *et al*.^80^ proposed that the *Symbiodinium* density increased because of an adaptation to low light conditions; in such conditions, the degradation rate of *Symbiodinium* decreased and was surpassed by the normal cell division rate of *Symbiodinium*, which finally resulted in the accumulation and increase in the number of *Symbiodinium* cells. However, this observation was in contrast to several studies^79,83^, which suggested that only the chlorophyll concentration is affected, and the *Symbiodinium* density remains unaffected. These contrasting views concerning the effect of light intensity and irradiance on the *Symbiodinium* density may also be dependent on other factors, such as water temperature and nutrient availability^43^, whereas other factors, such as salinity, starvation, photosynthetic active radiation, and osmotic shock, play a minor role in the *Symbiodinium* density. However, it will be important to characterize the light environments in these husbandry tanks, as well as perform more thorough photosynthesis-irradiance curve-based experiments, to know whether the cultured corals were truly not reaching their full photosynthetic potential at the light levels present in the culture tanks. It may be, for instance, that supplemental lights are needed.

Previously, we found that oocytes of the gorgonian coral *Junceella fragilis*, which, unlike those oocytes of the two coral species assessed herein, host *Symbiodinium*, contained significantly larger amounts of yolk bodies and lipid granules compared to the oocytes of the congeneric *Junceella juncea*, which does not harbor *Symbiodinium*^6^. If *Symbiodinium* indeed accumulate lipids when deprived of nitrogen, as mentioned above, it is possible that such accumulated lipids could be translocated to the oocytes; these lipids could then have contributed to the elevated levels of yolk material in oocytes containing *Symbiodinium* relative to those oocytes lacking *Symbiodinium*. If nitrogen-deprived *Symbiodinium* translocated accumulated lipids to the oocytes via the parent colonies in the cultured corals observed herein, then this might explain the high yolk densities in these samples. Muller-Parker *et al*.^78^ suggested that the translocation of lipids still continues even under low-nutrient conditions, since lipid droplets were still observed in endosymbiotic host coral gastrodermal cells even during nitrogen starvation

Mitochondria are inherited maternally and supply ATP to support oocyte development, spindle formation, cell division, fertilization, and meiosis^45,84–86^; hence, they are typically found in high densities in oocyte cytoplasm^68^. This was in contrast to our observation in which mitochondria were absent from cultured coral oocytes; in contrast, they were abundant, and quite large, in wild coral oocytes. Competent oocytes should feature mitochondrial densities sufficient for maintaining ATP levels for fertilization and blastocyst development^84^. Mitochondria have been reported to undergo transformation into yolk material^87,88^ in frogs^51^ and sea anemones^88^; if this phenomenon occurred in *O. lacera*, then this could explain both the absence of mitochondria and high yolk densities in the oocytes from cultured corals.

Offspring of certain coral genera, such as *Montipora, Porites*, and *Pocillopora*, inherit *Symbiodinium* from their parents^36,89^, a process known as vertical transmission; this phenomenon is more common in brooding corals. Broadcast spawners usually require their gametes or larvae to obtain *Symbiodinium* from the water column (i.e., horizontal transmission^90^). Inherited *Symbiodinium* in the coral oocytes may influence their settlement behavior and fertilization success, and certain *Symbiodinium* genotypes may be best suited for certain species^83^. *Symbiodinium* hosted in all of our sample coral colonies were identified as clade C1 (data not shown). Since oocytes of both target species were *Symbiodinium*-free, yet the host colonies all contained clade C *Symbiodinium*, we surmise that these species are horizontal transmitters. Horizontal transmission provides flexibility for the host to adapt under a wider range of conditions in the wild but could be detrimental in an aquaria featuring sand filtration, as the spawned oocytes would be unable to acquire *Symbiodinium*.

Herein we found that wild and cultured coral oocytes differed in their ultrastructure and physiology; notably, wild coral oocytes had thicker microvillus layers, and this may be related to the presumably higher potential for heterotrophy *in situ*. Cultured coral oocytes also contained significantly higher concentrations of yolk material, which could be indicative of an adaptive response to nutrient stress; large particles are removed from the seawater prior to its entering the husbandry tanks, and this might reduce the concentration of plankton and other coral food sources. These data will aid in future coral husbandry efforts, as well as in our cryopreservation studies. We had previously used wild oocytes only in our cryopreservation works^65,91^, though spawning occurs on only a few days in the summer and requires some fairly dangerous night dives; ideally, artificially reared corals could instead provide a more constant supply of oocytes, as they could be made to spawn at convenient times by modifying the light environment. However, given the differences in oocyte ultrastructure documented herein, it seems that cryotolerance may also prove to differ between wild and cultured coral oocytes, though this remains to be determined experimentally.

## Methods

### Collection for coral oocyte

Oocytes of both wild and cultured *O. lacera* and *E. gemmacea* were collected *in situ* (Houbihu, Pingtung, Taiwan, GPS: 218560 N, 1208440E) and in the husbandry facility of Taiwan’s National Museum of Marine Biology and Aquarium (NMMBA), respectively, in the April-May period of each of three years: 2014–2016. The exact spawning time is similar for the two species, though varied between years. Furthermore, the time of spawning was similar for cultured and wild corals (within 2–3 days) for each of the triplicate sampling years. Wild coral oocytes were collected at 3 to 5 m by SCUBA divers under Kenting National Park permit. The oocytes of both wild and cultured colonies (described below) were collected using a 50-mL syringe^92^ immediately after the oocyte bundles were released from the coral. For cultured *O. lacera* and *E. gemmacea*, coral colonies transported from the same study site to NMMBA’s husbandry center were cultured for at least seven years in a 5-ton open seawater tank under natural, shaded sunlight at 35 *psu*. The wild and cultured coral oocyte bundles of both species were then transferred in seawater immediately to the laboratory for washing with filtered seawater (0.4 µM filter, vacuum pump Rocker, 300, USA) and fixed in 1 hr after spawning for the subsequent ultrastructure observations. Both *in situ* and *ex situ* sample oocytes were collected on the most abundant of spawning day. *O. lacera* spawn almost simultaneous with *E. gemmacea* after sunset at between 1900 to 2000 hr. The spawning continues over 2 to 3 days. TEM observation and analysis was only conducted on eggs within the size (diameter) of 200–300 μm for uniformity. Based on our experience, the *O. lacera* and *E. gemmacea* spawn on the sixth or seventh night of the full moon. Thus this was used as the guide to determine the spawning timing of the corals. Apart from the physical parameters of the surrounding waters in the collection site of the *ex-situ* sample, light data time-series was not recorded. However a general impression of the *ex situ* sample collection site was that it is a tropical climate, and light availability is approximately 12 hours during April-May with the intensity peaking in the noon. With regards to seawater temperature, temperature was likely to fluctuate more than ± 3 °C within the range of 27–33 as oocytes of the two species were in collected in the photic zone ( < 10 m).

The light intensity of the culture tank was recorded every 15 min with a photometer for 45 hrs. As a partial outdoor husbandry facilities, the culture tank were able to received natural sunlight and no artificial light was used during the culture of the coral. Light intensity gradually increase from sunrise and peaked around noon (378.78 μmol m^−2^ s^−1^) and gradually decrease towards sunset at evening 6 o’clock.

### Species identification

A section of each coral colony was removed from the wild and cultured colonies and preserved in 30% sodium hypochlorite solution to dissolve the tissues. After 10 hrs of incubation, skeletons were washed in distilled water, dried, and subsequently observed under the light microscope (CX31, Olympus, Japan). Coral identification was performed to species level based on the polyp and sclerite morphology (corallite structure) *sensu* Dai and Horng^93^ and Lin *et al*.^94^.

### Preparation of oocytes for TEM

Isolated wild and cultured coral oocytes of each species were divided into (at least) 15-oocyte sub-samples for TEM. Oocytes were first fixed with 2.5% glutaraldehyde, 2% paraformaldehyde, 0.1 M phosphate buffer, and 5% sucrose for 2 hr and then post-fixed with 1% osmium tetroxide solution in the dark for 1 hr (All procedures were conducted in 4 °C). Oocytes were then washed thrice with 0.1 M phosphate buffer and shaken for 20 min on an orbital shaker (Major Science, Taiwan). Fixed oocytes then underwent gradual dehydration using 50, 70, 80, 90, 95, and 100% ethanol. TEM was conducted as described in Tsai *et al*.^70^. After serial dehydration, oocytes were incubated in 100% acetone. Next, infiltration and embedding were performed by placing the oocytes into plastic molds followed by addition of 100% ethanol with 100% Spurr’s resin at ratio of 1:1 for 1 hr, 1:3 for 1 hr, and finally only 100% Spurr’s resin for 24 hr. All procedures were conducted in 25 °C. Samples were placed in rubber moldings and then in an oven set at 60 °C for 48 h. Embedded samples were then allowed to polymerize. A diamond knife (MS 14410, Diatome, USA) secured on an ultramicrotome (Leica ultracut R, Leica, Germany) was used to section the polymerized blocks into 50–70 nm sections. Sectioned oocytes were collected and double-stained with 2% filtered uranyl acetate and then lead citrate (with 10 N NaOH). Samples were then observed with a Hitachi 600 TEM (JEM-1400, JEOL, Japan) at an accelerating voltage of 82 kV (filament, 56_A), and digital images were captured using a Gatan digital camera. ImageJ was used to assess the size of a variety structures, including microvilli, lipid granules (lg), yolk bodies (yb), and mitochondria (um).

### *Symbiodinium* density

Coral trusses were photographed under a stereomicroscope (Discovery Zeiss, Germany) and analyzed by ImageJ (National Institutes of Health of the USA). *Symbiodinium* were then isolated by adding 3% N-acetylcysteine (pH 8.2, Sigma-Aldrich, St. Louis, MO, USA) in artificial seawater, and a discontinuous sucrose gradient was used^70^ to obtain purified *Symbiodinium* cells. *Symbiodinium* cells were washed thrice with filtered seawater and resuspended in 1 mL 1X phosphate buffer. *Symbiodinium* density was determined in 100 μL aliquots with a Scepter handheld automated cell counter (EMD Millipore Corp., Billerica, MA, USA) and normalized to coral surface area. The remaining 900 μL of *Symbiodinium* cells were stored at −80 °C for further use. Obtaining of the genetic information of *Symbiodinium* was following the methods that we have done previously^8^.

### *Symbiodinium* protein and chlorophyll content

The *Symbiodinium* cells resuspended in PBS were homogenized using a TissueLyser LT (Qiagen, Hilden Germany) with glass beads, and protein content was analysed with a Pierce® BCA Protein Assay Kit (Thermo Scientific, Rockford, IL, USA) and normalized to dry tissue weight. The chlorophyll content of the *Symbiodinium* pellets was determined after extraction in acetone (final concentration of 90%) at 4 °C overnight in the dark. Absorbances of the samples, as well as that of the acetone blank, were analysed with a spectrophotometer (CT2200, ChromTech, MA, USA) at 630, 664, and 750 nm. Concentrations of chlorophylls a and c were then determined with the equations of Arar (1997):

1 $${\rm{Chl}}\,{a}({\rm{\mu }}g/{\rm{mL}})=(11.85\,{\rm{E}}664)-(0.08\,{\rm{E}}630)$$

2 $${\rm{Chl}}\,c({\rm{\mu }}g/{\rm{mL}})=(24.52\,{\rm{E}}630)-(1.67\,{\rm{E}}664)$$

where E630 = (A630sample − A630blank) − (A750sample − A750blank)

E664 = (A664sample − A664blank) − (A750sample − A750blank)

Chlorophyll a and c content (chl a and chl c, respectively) were normalized per cell (pg/cell).

### Water quality assessment

Water from the sampling site and the husbandry were collected and assessed to compare water quality. Quality control for the sampling equipment and field measurements were conducted in accordance with Taiwanese government QA/QC regulations. Water quality was assessed in a similar manner as Meng *et al*.^95^. Briefly, water temperature and salinity were measured *in situ* with a CTD (conductivity, temperature, and depth [pressure]) instrument (Sea-Bird Electronics Model 19 Plus). The precision for the temperature measurement was ± 0.05 °C, and the accuracy and precision for salinity were ± 0.003 and ± 0.023 *psu*, respectively. The water was maintained at 4 °C during transport from the field to the laboratory for analysis of the following parameters: pH, nutrient contents (nitrate, nitrite, phosphate, and ammonia), suspended solid levels, and water turbidity. A Flow Injection Analyzer (FIA) coupled with a spectrophometer (Hitachi model U-3000) was used to analyse the ammonia, nitrate, nitrite, phosphate, and silicate concentrations *sensu*^96^.

### Statistical Analysis

Three samples were prepared for each the wild and cultured coral species. Ultrastructural observations were repeated with three technical replicates from each of the three samples from species and treatment group (wild vs. cultured), and three sections were observed for each oocyte sample. Statistical analyses were conducted using SPSS (Ver.17) and Microsoft Excel. First, Kolmogorov-Smirnov tests was performed to ensure that all data were normally distributed, and one-way ANOVAs were used to test for the effect of husbandry (i.e., coral culture) on each of the response variables. Although the data from both species are presented together in the tables and figures, an effect of species was not included in the statistical models since we were interested only in the effect of husbandry on oocyte quality.
