# Author Correction: The effects of aquarium culture on coral oocyte ultrastructure

**DOI:** 10.1038/s41598-019-42138-5

**Published:** 2019-06-07

**Authors:** Chiahsin Lin, Jian-Ming Zhuo, Gabriella Chong, Li-Hsueh Wang, Pei-Jie Meng, Sujune Tsai

**Affiliations:** 1 National museum of Marine Biology & Aquarium, 2 Houwan Road, Checheng, Pingtung, 944 Taiwan; 2 Institute of Marine Biology, National Dong Hwa University, 2 Houwan Road, Checheng, Pingtung, 944 Taiwan; 3 Department of Biotechnology, Mingdao University, 369 Wen-Hua Road, Peetow, ChangHua, 52345 Taiwan; 4 Department of Post Modern Agriculture, Mingdao University, 369 Wen-Hua Road, Peetow, Chang Hua, 52345 Taiwan

Correction to: *Scientific Reports* 10.1038/s41598-018-33341-x, published online 11 October 2018

This Article contains an error in the legend of Figure 2.

“TEM images of *Oxypora lacera* mitochondria (m) and Golgi bodies (denoted by arrow) from wild (**a**) and cultured (**b**) oocytes. yb = yolk body. lg = lipid granule. The scale represents 1 um.”

should read:

“TEM images of *Oxypora lacera* mitochondria (m) and Golgi bodies (denoted by arrow) from wild oocytes (**a** and **b**). yb = yolk body. lg = lipid granule. The scale represents 1 um.”

In addition, this Article contains an error in the Discussions section.

“Herein we found that wild and cultured coral oocytes differed in their ultrastructure and physiology; notably, wild coral oocytes had thicker microvillus layers, and this may be related to the presumably higher potential for heterotrophy *in situ*.”

should read:

“Herein we found that wild and cultured coral oocytes differed in their ultrastructure and physiology; notably, wild coral oocytes had thinner microvillus layers, and this may be related to the presumably higher potential for heterotrophy *in situ*.”

